# *Euglossa obrima*, a new species of orchid bee from Mesoamerica, with notes on the subgenus *Dasystilbe* Dressler (Hymenoptera, Apidae)

**DOI:** 10.3897/zookeys.97.1106

**Published:** 2011-05-11

**Authors:** Ismael A. Hinojosa-Díaz, Gabriel A.R. Melo, Michael S. Engel

**Affiliations:** 1 Division of Entomology, Natural History Museum, and Department of Ecology & Evolutionary Biology, 1501 Crestline Drive–Suite 140, University of Kansas, Lawrence, KS 66049-2811, USA; 2 Univ. Fed. Parana, Dept. Zool., Lab. Biol. Comparada Hymenoptera, Caixa Postal 19020, BR-81531980 Curitiba, Paraná, Brazil; 3 Division of Invertebrate Zoology, American Museum of Natural History, Central Park West at 79th Street, New York, NY 10024-5192, USA

**Keywords:** Hymenoptera, Apoidea, Anthophila, Euglossini, new species, taxonomy, orchid bees

## Abstract

A new species of the orchid bee subgenus *Dasystilbe* Dressler (Euglossini: *Euglossa* Latreille) is described and figured from a series of males and females collected broadly in Mesoamerica. *Euglossa (Dasystilbe) obrima*, **sp. n.**, is differentiated from the one known species of *Dasystilbe*, *Euglossa (Dasystilbe) villosa* Moure, which occurs only in Panamá and perhaps Costa Rica. The subgenus and its constituent species are diagnosed, and comments provided on *Dasystilbe*.

## Introduction

Among corbiculate bees, the tribe Euglossini is famed for the characteristic morphological and behavioral features of its constituent species. In particular, their bright metallic coloration of the body and elongate mouthparts, and the collection and processing of fragrances by males, mainly from flowers of Orchidaceae, to which the group owes its common name – orchid bees. Of the five euglossine genera, *Euglossa* Latreille is the most diverse with about 120 species (Roubik and Hanson 2004, Moure et al. 2007), and six subgeneric names presently in use for their classification. Of the six subgenera, *Dasystilbe* Dressler is the most distinctive owing to its bizarre combination of morphological features that seemingly intermingle attributes of two of the other subgenera (*Euglossella* Moure and *Glossura* Cockerell), and the justification provided by Dressler (1978) for erecting this monotypic unit for *Euglossa villosa* Moure. The rather bizarre combination of characters and relatively restricted distribution of *Euglossa villosa*, combined by its unique status among all orchid bees, make *Dasystilbe* an interesting taxon for understanding euglossine phylogeny and biology. Most recently, phylogenetic analyses based both on molecular (Ramírez et al. 2010) and morphological (Hinojosa-Díaz 2010, in prep.) data place *Dasystilbe* in divergent positions, further stressing the complicated nature of these bees. However, the distinctiveness of *Euglossa villosa* is not exclusive to that species, and a second species of *Dasystilbe* is now known. Most importantly, females are described for the new species, while this sex remains unknown for *Euglossa villosa*.

Herein we provide a brief, illustrated account of the subgenus, as well as the description of a new, second species, including females, and a comparative diagnosis for *Euglossa villosa*.

## Material and methods

Material used in this study is deposited in the following collections: Division of Entomology, University of Kansas Natural History Museum, Lawrence, Kansas, USA; Museo de Zoología Alfonso L. Herrera, Facultad de Ciencias, Universidad Nacional Autónoma de México, México, D.F., Mexico; Florida Museum of Natural History, University of Florida, USA; and the Departamento de Zoologia, Universidade Federal do Paraná, Curitiba, Paraná, Brazil.

Morphological terminology generally follows that of Engel (2001), Michener (2007), and Hinojosa-Díaz (2008), while the overall format of the descriptions mirrors that used elsewhere for species of *Euglossa* (e.g., Hinojosa-Díaz and Engel 2007). Photomicrographs were prepared using a Nikon D1x digital camera attached to an Infinity K-2 long-distance microscopic lens.

## Systematics

### Genus Euglossa Latreille

#### Subgenus Dasystilbe

Dressler

http://species-id.net/wiki/Euglossa_(Dasystilbe)

Euglossa (Dasystilbe)  Dressler, 1978: 193. Type species: *Euglossa villosa* Moure, 1968, by original designation.

##### Diagnosis.

**Figures 1–3.**
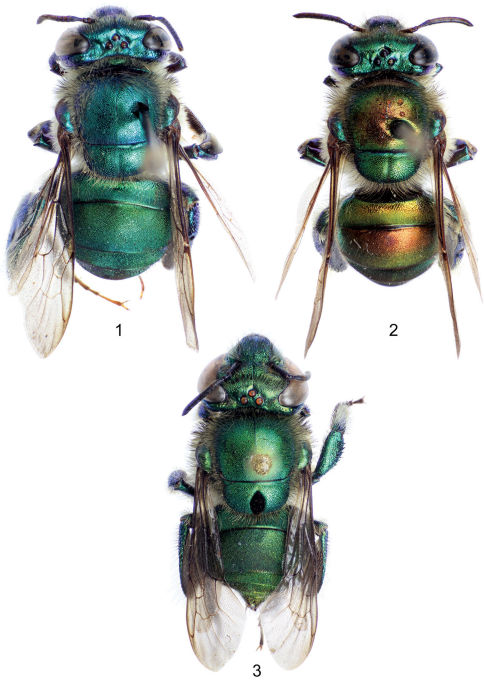
Dorsal habitus of species of *Euglossa (Dasystilbe)*. **1** *Euglossa (Dasystilbe) obrima*, sp. n., male holotype **2** *Euglossa (Dasystilbe) villosa* Moure, male **3** *Euglossa (Dasystilbe) obrima*, sp. n., female paratype.

Large bees (body length nearly 15 mm), body coloration bright metallic green with either bronzy or blue iridescence and noticeable differently-colored apical bands on first four metasomal terga (Figs 1–3); body covered with noticeable long dense fulvous setae especially on lateral and ventral sides; upper and lower interorbital distances equal; clypeus not noticeably protuberant (no more than 0.90 mm); labrum rectangular, wider than long; male mandible bidentate, female mandible tridentate; labiomaxillary complex in repose reaching at most posterior margin of second metasomal sternum; pronotal dorsolateral angle projected laterally as an acute prong (Fig. 10); posterior border of mesoscutellum semi-ellipsoidal, female with a large dark mesoscutellar patch (Fig. 3); male mesotibia with two setose patches, anterior one large, ellipsoidal, occupying nearly one-third of mesotibial length, posterior one ovoid-oblong about one-third as long as anterior patch (Figs 15–16); microtrichia of velvety area on male mesotibia becoming sparser anteriorly (Figs 15–16); inner surface of male mesobasitarsus with a prominent distal elevation obliquely ridged (Fig. 11); second mesotarsomere of male with basal emarginations on both anterior and posterior margins (this may sometimes be obscured by expansion of the inner surface); male metatibia triangular with no evident furrow on posterodorsal margin; metatibial organ slit not reaching ventral margin of male metatibia, but separated from it by less than its own length (Figs 19–20); male metabasitarsus lanceolate, anterior margin conspicuously convex especially on proximal section (Fig. 12); metafemur of male with ventral margin strongly concave especially as seen on inner surface (Fig. 14); male second metasomal sternum with two cowled slits [*sensu* Roubik (2004)] posteriorly narrowed and separated by about one and a half times width of an individual slit (Fig. 13); eighth metasomal sternum with noticeable lobes on lateral margins (Fig. 22), with posterior section of eighth metasomal sternum laterally about as wide as lateral width of anterior section (Fig. 23); dorsal process of gonocoxite noticeably longer than wide; lateral section of gonostylus with concave inner setose area covered by long simple setae (Figs 24–26).

##### Euglossa (Dasystilbe) obrima

Hinojosa-Díaz, Melo, & Engel sp. n.

urn:lsid:zoobank.org:act:A0BB5B62-C63B-46C5-900D-DB7AD9FB5FA0

http://species-id.net/wiki/Euglossa_(Dasystilbe)_obrima

Figs 1, 3 4, 6 7, 9 10 15, 17, 19 21–26 

###### Holotype.

**Figures 4–6.**
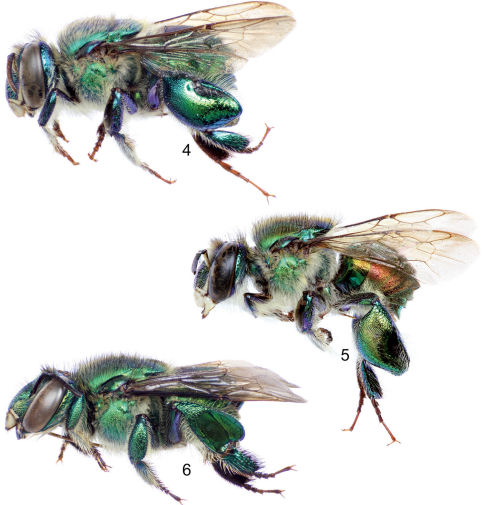
Lateral habitus of species of *Euglossa (Dasystilbe)*. **4** *Euglossa (Dasystilbe) obrima*, sp. n., male holotype **5** *Euglossa (Dasystilbe) villosa* Moure, male **6** *Euglossa (Dasystilbe) obrima*, sp. n., female paratype.

**Figures 7–9.**
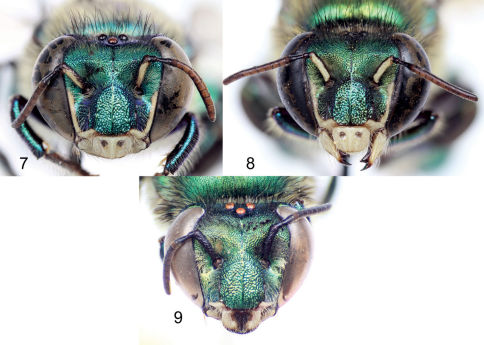
Facial aspect of species of *Euglossa (Dasystilbe)*. **7** *Euglossa (Dasystilbe) obrima*, sp. n., male holotype **8** *Euglossa (Dasystilbe) villosa* Moure, male **9** *Euglossa (Dasystilbe) obrima*, sp. n., female paratype.

 ♂ (Figs 1, 4, 7), labeled, “Mexico, VeraCruz; 34 km N Catemaco; UNAM Reserve; Jan. 6, 1982; John W. Wenzel // Euglossa; villosa Moure; Det. R.L. Dressler, 1987”. The holotype is in the Division of Entomology, University of Kansas Natural History Museum, Lawrence, Kansas, USA.

###### Paratypes.

16♂♂, 13♀♀: labeled as follows: data as holotype except date “Jan. 10, 1982” (1♂); “MEXICO: Vera-; cruz. Fortin; 4 XI 1966 [date handwritten except three first digits of year]; R.L. Dressler 591 [number handwritten]” (1♂); “MEXICO, Veracruz; Teocelo; 25-VIII-1989; 12:00 1080m; A. Callejas” (1♀); Chalchijapa, Santa María; Chimalapa, Oaxaca. [Mexico]; 22-III-1995; J.L. Salinas 100 m; Selva Alta Perennifolia; Atraida con Esencias JL-183; Galera Rolando 08:28 h // MUSEO DE ZOOLOGIA; HYMENOPTERA; 10576” (1♂); same data except last number in second label “10583” (1♂); “10585” (1♂); same data except: date “24-III-1995”, last two lines on first label “Atraida con Esencias JL-191; Campamento 12:48 h”, last number in second label “10631” (1♂); same data except: date “26-III-1995”, last two lines on first label “Atraida con Esencias JL-194; 10:00 h”, last number in second label “10646” (1♂); same data except: date “24-VIII-1995”, last two lines on first label “Atraida con Esencias RL-1007; 12:30 h”, last number in second label “11308” (1♀); “MEXICO, Hidalgo; Tlanchinol 1516 m; Tlanchinol-Apantlazol Km 1; 29[20°?] 59’13” N 98[°] 39’04” W; 14-09-1993 10:45 Hrs.; L. Godínez LG-898” (1♂); same data except last two lines: “14-09-1993 12:30 Hrs.; R. López RL-81” (1♂); same data except last two lines: “05-06-1993 11:00 Hrs.; L. Godínez LG-787” (1♀); “MEXICO, Hidalgo; Tlanchinol 1600 m; Tlanchinol-Apantlazol Km 4; 20[°] 59’04” N 98[°] 38’13” W; 13-09-1993 10:20 Hrs.; R. López RL-76” (1♂)”; “Museo de Zoología; Fac. De Ciencias; U.N.A.M. // MEX[Mexico]: Oax.[Oaxaca]; Metates.; 13-IX-1987 // MUSEO DE ZOOLOGIA; HYMENOPTERA; 10441” [second label handwritten] (1♀); “Museo de Zoología; Fac. De Ciencias; U.N.A.M. // MEX[Mexico]: Oax.[Oaxaca]; Macultianguis [Macuiltianguis?]; 17-IX-1987; A. Luis // MUSEO DE ZOOLOGIA; HYMENOPTERA; 10422” [second label handwritten] (1♀); “Museo de Zoología; Fac. De Ciencias; U.N.A.M. // Edo. Oax.,[Oaxaca, Mexico] Pto; Eligio. Mpio.; Comaltepec; 600 msnm.; 9-VIII-1986, A. Luis // MUSEO DE ZOOLOGIA; HYMENOPTERA; 10421” [second label handwritten] (1♀); “MEXICO: San Luis; Potosi, El Limon 70 km S; Ciudad Valles on Hwy 85; RL Minckley & BN; Danforth 29–30 Dec 1988 // Euglossa villosa [handwritten]; Moure; det. R.W.Brooks 1996 [last two digits handwritten]” (1♂, 1♀); “MEXICO: San Luis; Potosi, San Juan, 16 Feb.; 1992, L. Godinez, #818; *ex Bidens odorata* // Euglossa [handwritten]; villosa Moure [handwritten]; det. R.W.Brooks 1996 [last two digits handwritten]” (1♀); “MEXICO: San Luis; Potosi, Xilitla, 1km E; Xilitla at river 400m; 9 July 1990, I. Yarom” (1♀); “Atoyac,; Vera Cruz.; Schumann// Godman-Salvin; Collection.; 1913-214.// Euglossa; villosa ; m. [handwritten]; Det. J.S. Moure 1958 [last two digits handwritten]” (1♂); “MEX[Mexico]: S.[San] L.[Luis] P.[Potosi]; Tamazunchale; IV-12-54// D.H. Janzen; Collector” (1♂); “Finca’ La Isle’; Chiapas, Mex.[Mexico]” (1♀); “4 mi[miles] NW Ocosingo; Chiapas. MEX.[Mexico]; III-8-1953” (1♀); “San Juan,; Vera Paz; Champion.// Godman-Salvin; Collection.; 1913-214.// viridissima; Friese [handwritten]; Pe J. S. Moure 1968 [last two digits handwritten] [underside of label: MUS. HUNGARICUM; C.[compared] W.[with] T.[type] ♀; MEXICO; Ctba.[Curitiba] 29-XI-1968 [handwritten]]” (1♀); “GUATEMALA: Zacapa; Prov., 3.5 km SE La Union; 1500m, 25–27 June 1993; J.Ashe&R.Brooks#128; *ex*:flight intercept trap // Euglossa [handwritten]; villosa Moure [handwritten]; det. R.W.Brooks 19” (1♂); “La Conquista; Guatemala// Euglossa ♀; villosa m. [handwritten]; Pe J. S. Moure 1968 [last two digits handwritten]” (1♀); “Honduras: Santa Barbara; Finca Las Quebradas, W; of Lake Yojoa, Oct. 1991” (1♂); “Jinotega; NICARAGUA; C.H. Dodson [underside of label: Sept. 1963]// HOLOTYPUS [labelled as a holotype by Moure but never published]; Dasystilbe [plus male symbol]; smaragdula [handwritten]; Pe. J. S. Moure 1985 [last two digits handwritten]” (1♂). Paratypes are deposited in the Division of Entomology, University of Kansas Natural History Museum, Lawrence, Kansas, USA; Museo de Zoología Alfonso L. Herrera, Facultad de Ciencias, Universidad Nacional Autónoma de México, México, D.F., Mexico; Florida Museum of Natural History, University of Florida, USA; and Departamento de Zoologia, Universidade Federal do Paraná, Curitiba, Paraná, Brazil.

###### Diagnosis.

Bees with a rather stocky habitus, both sexes with body coloration bright metallic green, with faint bronzy hue and blue iridescence, posterior section of first four metasomal terga with noticeable cyan-blue iridescence forming a band along tergal margins (Figs 1, 3); punctation moderately dense; body with dense, fulvous, long setae especially on lateral and ventral sides of head and mesosoma; female with conspicuous ellipsoidal setal patch on mesoscutellum made of dense, dark setae (Fig. 3); male with mesotibial anterior tuft ellipsoidal with a diagonally truncate base and a distal rounded margin, posterior tuft round, oblong partially lying on posterior half of truncate margin of anterior tuft, velvety area noticeably sparser along anterior mesotibial margin (Fig. 15); mesotarsomeres beyond mesobasitarsus longer than wide, especially second mesotarsomere (Fig. 17); distal section of metatibial organ slit lanceolate (spur-like), slender (maximum width occupying about one-fifth of metatibial outer surface width) (Fig. 19); second metasomal sternum with two narrow cowled slits (Fig. 13).

###### Description.

**Figures 10–14.**
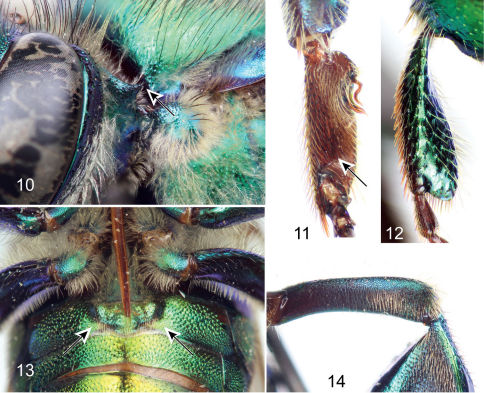
Diagnostic features of the subgenus *Dasystilbe*, as observed on male specimens of *Euglossa (Dasystilbe) obrima*, sp. n. **10** Acute projection of pronotal dorsolateral angle (arrow) (some setae were removed to expose this feature) **11** Ridge on inner surface of mesobasitarsus (arrow) **12** Metabasitarsus **13** Cowled slits on second metasomal sternum (arrows) **14** Metafemur.

♂: *Structure*. Total body length 13.54 mm (12.30–14.67; n=5); labiomaxillary complex in repose reaching sternum III (not exceeding it) (Fig. 13). Head length 3.11 mm (2.96–3.33; n=5), width 5.19 mm (5.11–5.26; n=5); upper interorbital distance 2.31 mm (2.30–2.37; n=5); lower interorbital distance 2.29 mm (2.22–2.37; n=5); upper clypeal width 1.39 mm (1.26–1.48; n=5) (as measured between dorsolateral angles of clypeus); lower clypeal width 2.22 mm (2.19–2.30; n=5) (as measured at level of lower lateral parts); clypeal protuberance 0.76 mm (0.74–0.81; n=5) (following measurement method of Brooks 1988); medial clypeal ridge sharp, paramedial clypeal ridges not as sharp but well developed; labrum rectangular, wider than long, length 1.09 mm (1.04–1.11; n=5), width 1.27 mm (1.26–1.33; n=5) (Fig. 7); medial labral ridge sharp; paramedial labral ridges not as sharp, oblique, well developed along entire labral length; labral windows ovoid, occupying proximal one-half of labrum; interocellar distance 0.35 mm (0.30–0.41; n=5); ocellocular distance 0.67 mm (n=5); first flagellomere nearly as long [0.52 mm (n=5)] as second and third flagellomeres combined [0.50 mm (0.48–0.52; n=5)]; length of malar area 0.13 mm (0.11–0.15; n=5). Mandible bidentate. Dorsolateral angle of pronotum projected laterally as an acute prong (Fig. 10); intertegular distance 3.94 mm (3.85–4.07; n=5); mesoscutal length 3.19 mm (3.07–3.41; n=5); mesoscutellar length 1.50 mm (1.48–1.56; n=5); posterior border of mesoscutellum demi-ellipsoidal (Fig. 1); mesotibial length 2.68 mm (2.59–2.74; n=5); mesobasitarsal length 2.36 mm (2.30–2.37; n=5), width 0.84 mm (0.81–0.89; n=5) (as measured at proximal posterior keel), posterior keel projected in a right or slightly acute angle, inner surface with a prominent distal elevation obliquely ridged forming concavity contiguous to emarginated joint with second mesotarsomere (Fig. 11); mesotarsomeres beyond mesobasitarsus longer than wide, especially second mesotarsomere in which posterior margin is conspicuously concave (Fig. 17); metatibial shape triangular (scalene right triangular); metatibial anterior margin length 3.84 mm (370–3.93; n=5), ventral margin length 3.19 mm (2.89–3.33; n=5), postero-dorsal margin length 4.97 mm (4.74–5.19; n=5), maximum thickness 1.44 mm (1.41–1.48; n=5); metatibial organ slit basal and distal sections well defined with junction nearly as wide as contiguous width of basal section; distal section of metatibial organ slit lanceolate (spur-like), maximum width occupying about one-fifth of metatibial outer surface width (Fig. 19); dorsal section of metatibial organ slit rhomboid, length 0.62 mm (0.52–0.81; n=5); metabasitarsal length 2.78 mm (2.67–2.96; n=5), mid-width 0.98 mm (0.89–1.04; n=5); metabasitarsal ventral margin projected posteriorly on a rounded slightly obtuse angle. Forewing length 10.40 mm (10.07–10.89; n=5); jugal comb with 13–15 (n=5) blades; hind wing with 22–28 (n=5) hamuli. Maximum metasomal width 5.54 mm (5.41–5.70; n=5); second metasomal sternum as described for subgenus (Fig. 13).

*Coloration*. Head bright metallic green (except as described below), with faint bronzy hue, specially on midclypeus, and blue iridescence adjacent to torulus; paraocular ivory marks well developed, slightly wider basally (around half distance to lateral margin of clypeal disc), inner margin of marks irregular; labrum ivory; labral anterior and posterior edges as well as labral windows amber-translucent; malar area metallic-green on condyle, brown on acetabulum, ivory in between; mandible ivory on about two thirds of outer surface, teeth and ridges brown; antenna brown, lighter on posterior surface of flagellum; scape with ivory spot covering entire anterior surface (Fig. 7). Mesosoma bright metallic green with faint bronzy hue (darker and more evident on anteriorly oriented episternal surface), blue highlights specially along sutures and sulci (Figs 1, 4); legs metallic-green on outer surface of all major podites, darker and duller than mesosoma (except meta-leg), with same combination of faint bronzy hue and blue highlights on edges, inner surfaces of most podites and most integument of meta- and mesotrochanters as well as all tarsomeres beyond mesobasitarsus, shiny brown combined with blue-green iridescence (Figs 14, 17); tegula colored as rest of mesosoma, wings bright amber, with brown veins and stigma (Figs 1, 4). Metasomal terga bright metallic green, noticeable bronzy-gold iridescence on anterior section of first four terga and on entire surface of the remainder ones, posterior section of first four terga with noticeable cyan-blue iridescence band along tergal margins, slightly wider mesally, covering about half of dorsal surface of first tergum and about one-third of second to fourth tergal dorsal surfaces (Fig. 1), ventro-lateral sections of first metasomal tergum with amber glow. Sterna metallic-green with strong bronzy-gold iridescence especially on narrow anterior sections and posterior margins, sometimes turning into amber-brown glow, especially noticeable on first sternum and on cowled slits of second sternum (Fig. 13).

**Figures 15–20.**
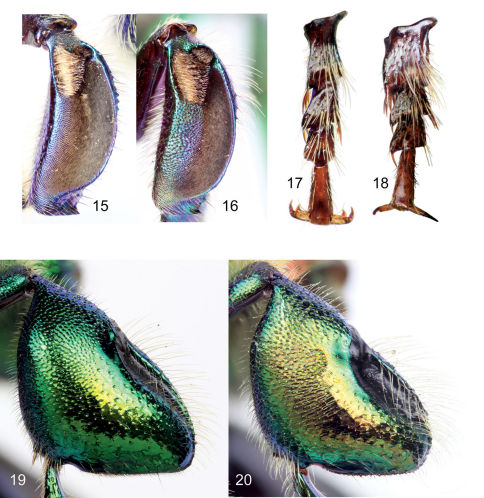
Features to differentiate males of the two species of subgenus *Dasystilbe*. **15** Mesotibia of *Euglossa (Dasystilbe) obrima*, sp. n. **16** Mesotibia of *Euglossa (Dasystilbe) villosa* Moure **17** Mesotarsomeres (excluding mesobasitarsus) of *Euglossa (Dasystilbe) obrima*, sp. n. **18** Mesotarsomeres (excluding mesobasitarsus) of *Euglossa (Dasystilbe) villosa* **19** Metatibia of *Euglossa (Dasystilbe) obrima*, sp. n. **20** Metatibia of *Euglossa (Dasystilbe) villosa*.

*Sculpturing*. Frons areolate, with dense, strong punctures (rather areole-punctures) small (diameter about one tenth of mid-ocellus diameter), increasing in size towards lower facial sections, especially on clypeal disc between paramedial ridges (punctures two to three times as large as on frons), puncture density diminishing on paraocular areas (punctures not contiguous), leading to narrow smooth integumental areas on antennal depression around torulus, and on a deep, narrow groove between torulus and frontal line; ivory areas (paraocular marks, clypeal lateral portions, mandibles and labrum) with shallow sparse punctures; vertex with moderately dense, shallow, small punctures on posterior margin, and interocellar area, areas of smooth, shiny integument present on anterior section of mid-ocellus and antero-lateral sections of lateral ocelli, smooth (minutely rugulose), dull integument on ocello-ocular groove (Fig. 1); posterior half of gena sculptured as posterior section of vertex, anterior half with sparse minute punctures, and scattered, large, round punctures along compound eye. Mesoscutum moderately punctate, round punctures about one-tenth of mid-ocellus diameter, separated by about half to one puncture diameter, puncture size slightly increasing on narrow anterior margin; mesoscutellum with similar general pattern as mesoscutum, specially on anterior half (except rather smooth along anterior margin), puncture size gradually increasing towards posterior margin such that posteriormost punctures are nearly four times as large as those on anterior half, except on mid groove where punctures are denser (contiguous) and of same size as those on anterior mesoscutellar half; mesepisternum punctation as that on mesoscutum, except larger, denser punctures on uppermost section; major leg podites dense to moderately dense punctate on outer surfaces, punctures generally shallow, punctures on metatibia dense (contiguous) on upper anterior margin, puncture size increasing, and density decreasing towards posterior and ventral margins, such that there is rather smooth integument along tibial organ slit and ventral margin. Metasomal terga in general densely punctate, dorsal surface of first tergum with relatively large punctures (as large as those on posterior margin of mesoscutellum) on anterior half especially on mesal area, punctures on posterior portion slightly smaller than those on frons or on mesoscutum, ventrolateral sections polished smooth; second to fourth terga with dense punctures equivalent in size to those on frons or on mesoscutum, only slightly smaller towards posterior margin along the characteristic cyan-blue iridescence bands, these three terga with larger and not as dense punctures on ventrolateral margins; anterior half of dorsal surface and lateral area of fifth to seventh terga with moderately dense large punctures as large as those on posterior margin of mesoscutellum, posterior mesal half of these three terga with punctation equivalent to that on second to fourth terga; metasomal sterna densely punctate, punctures as large or larger than those on posterior margin of mesoscutellum, decreasing slightly in size towards posterior margin of each sternum, although more noticeably on second sternum, lower puncture density on anterior margin and on medial body line.

*Vestiture*. Frontal fringe composed of two kinds of moderately dense setae, fulvous, plumose (moderately long branches), rather long (length around one and a half mid-ocellus diameter), setae covering the lower anterior area of the fringe, and dark brown, minutely branched (appearing simple), slightly longer, sturdier, setae long, both intermixing midway on the fringe; clypeus, paraocular area, antennal depression, labrum, mandible and malar area, with moderately dense, fulvous setae similar to those on lower half of frontal fringe, except as follows: appressed and noticeably plumose (long branches) on antennal depression, less dense, and with very short branches (appearing simple) on clypeus disc, labrum, mandibles and malar area, these last also having intermixed some scattered brown simple setae; vertex with some scattered, fulvous short, plumose setae on ocello-ocular groove and posterior area of ocellar triangle; dark setae of the same nature as those on posterior half of frontal fringe scattered (but slightly longer) on interocellar area, ocello-ocular groove, and posterior margin, where density increases and they intermix with fulvous setae similar to those on lower half of frontal fringe; gena with dense, fulvous, setae, short and simple on upper section and becoming longer and with longer branches toward lower genal section. Prothorax with setae as those on lower half of frontal fringe, intermixed with the other kind of setae on pronotal lobe; mesoscutum and mesoscutellum covered with a combination of dense setae of the same nature as the two kinds on frontal fringe, slightly longer towards anterior margin of mesoscutum and posterior margin of mesoscutellum, on this last area the fulvous setae are minutely branched and rather appearing as the dark ones except different coloration; mesepisternum and entire lateral mesosomal areas covered with dense, fulvous, moderately long, plumose setae, with very few scattered dark, hirsute setae on uppermost mesepisternal areas (Fig. 4); all coxae and trochanters with vestiture agreeing with that of mesepisternum, except for mesotrochanter with a rather bare anterior surface, and ventral surface with a particular patch of appressed, short, simple setae contiguous to femur joint; inner surface of all basitarsi with dense, brown, simple, strong setae, pointing ventrally, additionally, mesobasitarsal inner surface with three major wavy setae arising along edge of proximal keel; posterior surface of profemur covered with dense, fulvous, plumose, setae, proximally about as long as those on lower gena section, becoming shorter distally; posterior surface of protibia and outer surface of probasitarsus with similar setae, however not as plumose, anterior and inner surface of profemur and protibia with moderately dense, fulvous, simple, short setae; distalmost margin of probasitarsus and second and third protarsomeres with chemical gathering tufts composed of tightly dense, reddish-ochre, simple, long setae; outer surface of mesotibia with two proximal tufts, anterior tuft ellipsoidal with a diagonally truncate base and rounded on its distal end (Fig. 15), about one-third as long as velvety area, posterior tuft round, oblong, about one-third as long as major axis of anterior tuft, and partially lying on posterior half of truncate margin of anterior tuft (Fig. 15), both tufts on deep cavities, and composed of dense, fulvous, plumose setae directed posterad, longer on anterior tuft; microtrichia of velvety area covering the remainder outer surface, although noticeably sparser along anterior margin (Fig. 15); outer surface of metafemur, metatibia and metabasitarsus with moderately dense, pale, rather simple (minutely branched) setae, as long as those on frontal fringe, especially on metafemur, slightly shorter on metabasitarsus and metatibia, except this last with a fringe of enlarged setae on postero-dorsal margin along distal section of organ slit, some of these setae appressed over organ slit; metatibial organ slit closed with dark brown setae (Fig. 19). Dorsal surface of first metasomal tergum covered with moderately dense, fulvous, rather simple setae as long as those on frontal fringe, ventro-lateral sections bare; second to fourth metasomal terga covered with dense, fulvous, simple, very short setae all throughout, intermixed with scattered, dark-brown, erect, simple, short, setae especially on dorsum towards posterior margin; fifth to seventh terga with setae as those on dorsum of first tergum; first metasomal sternum mesally with a dense patch of fulvous, plumose, appressed, long setae; all remaining sterna with vestiture as that on dorsum of first metasomal tergum; cowled slits on second sternum covered with moderately dense, pale, simple, setae oriented posterad, and also forming a fringe along edge of openings (Fig. 13).

**Figures 21–26.**
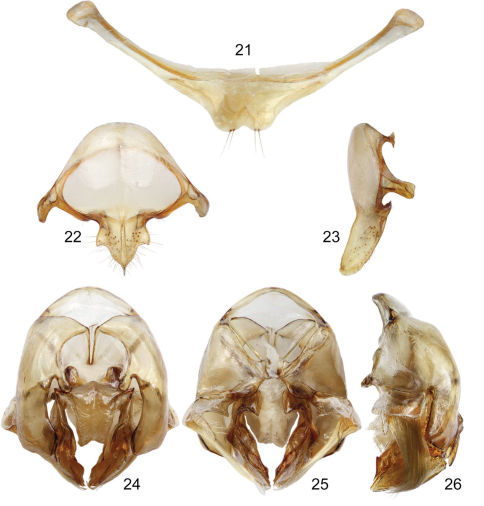
Male genitalic features of *Euglossa (Dasystilbe) obrima*, sp. n. **21** Seventh metasomal sternum, ventral aspect **22** Eighth metasomal sternum, ventral aspect **23** Eighth metasomal sternum, lateral aspect **24** Genitalic capsule, dorsal aspect **25** Genitalic capsule, ventral aspect **26** Genitalic capsule, lateral aspect.

*Terminalia*. Posterior margin of disc of seventh metasomal sternum invaginated mesally, with two apical setae on each side of invaginated section (Fig. 21). Eighth metasomal sternum with anterior section wider than long (not considering apodemes); posterior section projected ventrally making an angle of ~160° respect to longitudinal axis of anterior section; lateral lobes of posterior section with lateral edge slightly convex and an acute point posteriorly, some rather long setae inserted mainly on ventral surface of lobes; lateral width of posterior section comparable to lateral width of anterior section (without apodemes) (Fig. 23). Dorsal process of gonocoxite longer than wide; incision between posterior and dorsal processes of gonocoxite forming an acute angle (Fig. 24). Gonostylus with a lateral section projected beyond the margin of gonocoxite (in dorsal or ventral views), bearing dense, fulvous, simple, long setae on concave inner surface (setae slightly surpassing posterior margin of blades of penis valves); ventral lobe of gonostylar lateral section triangular, bearing some scattered setae on inner surface (Figs 24–26).

♀: *Structure*. Total body length 12.87 mm (12.44–13.56; n=5); labiomaxillary complex in repose reaching posterior margin of first metasomal sternum. Head length 3.31 mm (3.19–3.41; n=5); head width 5.27 mm (5.19–5.48; n=5); upper interorbital distance 2.54 mm (2.50–2.59; n=5); lower interorbital distance 2.49 mm (2.44–2.63; n=5); upper clypeal width 1.36 mm (1.33–1.41; n=5); lower clypeal width 2.30 mm (2.22–2.37; n=5); clypeal protuberance 0.86 mm (0.74–0.89; n=5); medial clypeal ridge sharp, paramedial ridges sharp on lower half; labrum rectangular, wider than long, length 1.11 mm (n=5), width 1.34 mm (1.31–1.41; n=5) (Fig. 9); labral ridges and windows as in male; distal margin of labrum with a submarginal carina produced outwards; interocellar distance 0.37 mm (0.36–0.37; n=5); ocellocular distance 0.74 mm (0.73–0.78; n=5); first flagellomere nearly as long [0.53 mm (0.52–0.58; n=5)] as second and third flagellomeres combined [0.50 mm (0.48–0.52; n=5)]; length of malar area 0.13 mm (0.11–0.15; n=5). Mandible tridentate, basal tooth broader than other two teeth. Dorsolateral angle of pronotum as in male; intertegular distance 3.99 mm (3.63–4.07; n=5); mesoscutal length 3.29 mm (3.11–3.41; n=5); mesoscutellar length 1.56 mm (1.52–1.63; n=5); posterior border of mesoscutellum as in male (Fig. 3; mesotibial length 2.73 mm (2.67–2.96; n=5); mesobasitarsal length 2.33 mm (2.15–2.44; n=5), maximum width 0.75 mm (0.74–0.81; n=5); metatibia triangular; metatibial anterior margin length 3.96 mm (3.78–4.15; n=5), ventral margin length 2.28 mm (2.22–2.44; n=5); postero-dorsal margin length 4.29 mm (4.19–4.52; n=5); metatibial ventro-posterior angle evenly rounded. Forewing length 10.00 mm (9.63–10.81; n=5); hind wing with 24–27 (n=5) hamuli. Maximum metasomal width 5.61 mm (5.48–5.85; n=5).

*Coloration*. As described for the male except as follows: paraocular marks and spot on antennal scape absent; ivory coloration on outer surface of mandible covering no more than one-third of it; labrum with distal margin dark brown to black, mid surface between paramedial ridges with a noticeably dark brown spot (Fig. 9).

*Sculpturing*. As described for the male except mesepisternum slightly denser all over and no major puncture size distinction on upper section. Metatibial corbicular concavity smooth.

*Vestiture*. As described for the male except as follows: mesoscutellar patch oblong, composed of dense, dark, erect setae with some pale setae intermixed, length of patch occupying about three-quarters of mesoscutellar length, and width of patch about one-fifth of mesoscutellum (Fig. 3). Mesotibia with some spur-like, dark brown setae on posterior and ventral edges; metatibial corbicula surrounded by long, pale setae, and near anterior margin with a few sturdy simple dark setae. Mesal sections of second to fourth metasomal sterna nearly bare.

###### Distribution.

**Figure 27.**
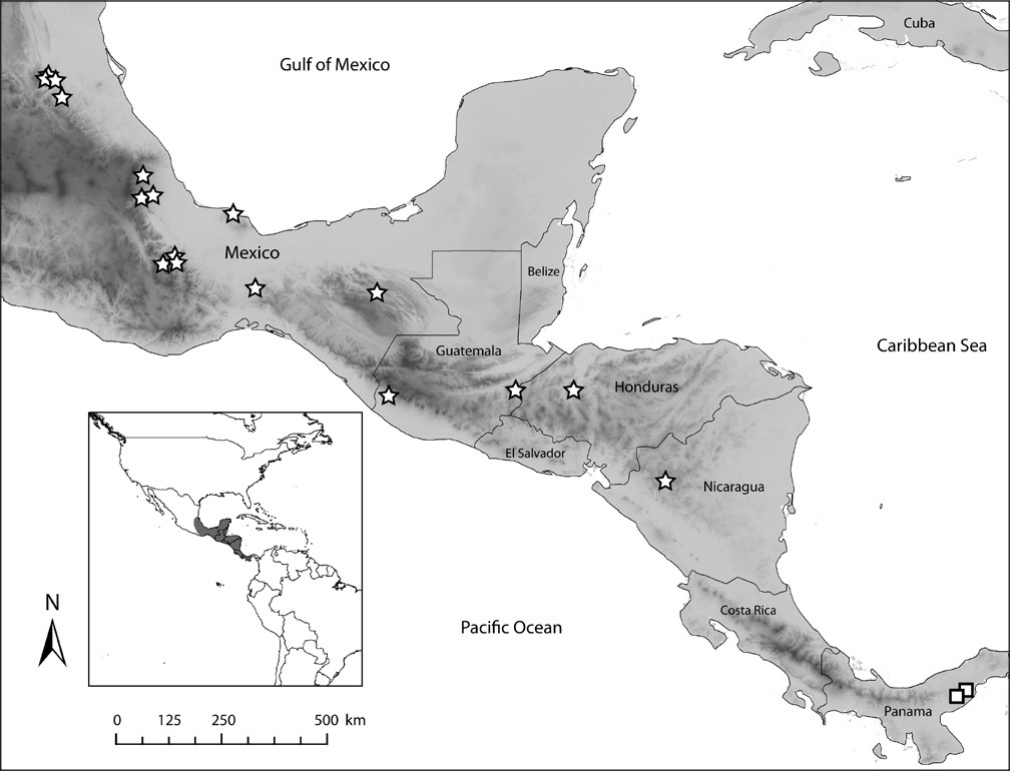
Distribution of the two species of *Euglossa (Dasystilbe)*. Stars indicate localities for *Euglossa (Dasystilbe) obrima* sp. n., squares indicate localities for *Euglossa (Dasystilbe) villosa* Moure.

 Known from as far south as Nicaragua, and as north as the central Mexican state of San Luis Potosí (Fig. 27). *Euglossa obrima* is found in humid environments with specimens collected in lowland areas with tropical rain forest (Chimalapas, Oaxaca, Mexico), or at mid altitudes (as high as 1600 m) in cloud mountain forest (Tlanchinol, Hidalgo, Mexico). Although collecting records are scarce, beyond the Isthmus of Tehuantepec going north, the species is absent from the Mexican lowlands along the Pacific Ocean, with records only from the lowlands along the Gulf of Mexico. The distribution of *Euglossa obrima* is apparently disjunct to the known distribution of *Euglossa villosa* which is known only from a couple of localities in Panamá, although some specimens missing locality data, deposited in the Florida Museum of Natural History, University of Florida, USA, may be from Costa Rica (Mark Whitten, pers. comm. 2006).

###### Comments.

 The males collected in Chalchijapa, Santa María Chimalapa, Oaxaca, Mexico as well as those from Tlanchinol, Hidalgo, Mexico, were captured using a mix of chemicals including eugenol and methyl salicylate. One male from Guatemala was captured in a flight intercept trap, while one female from San Juan, San Luis Potosi, Mexico was collected while visiting flowers of *Bidens odorata* Cavanilles (Asteraceae).

###### Etymology.

 The specific epithet is a reference to the slightly broader general habitus of this species when compared to *Euglossa villosa* (Greek, *obrimos*, meaning “strong”, “mighty”).

##### Euglossa (Dasystilbe) villosa

Moure

http://species-id.net/wiki/Euglossa_(Dasystilbe)_villosa

Figs 2 5 8 16, 18, 20 

###### Diagnosis.

Only males presently known; body coloration metallic-green with noticeable bronzy iridescence particularly on mesoscutum, metatibia, and metasoma (Figs 2, 5, 20); first four metasomal terga with noticeable green band along tergal margins as opposed to their conspicuously bronzy iridescent major anterior sectors (Figs 2, 5); punctation moderately dense; body with dense, fulvous, long setae especially on lateral and ventral sides of head and mesosoma; mesotibial anterior tuft ellipsoidal with a diagonally truncate base and also a truncate distal margin, posterior tuft oval, almost completely lying on two-thirds of truncate proximal margin of anterior tuft, sparse section of velvety area covering about anterior half of it (Fig. 16); second mesotarsomere quasi square-shaped, posterior margin concavity obscured by projection of posterior margin of inner surface (Fig. 18); third and fourth mesotarsomeres about as long as wide or slightly wider; distal section of metatibial organ slit lanceolate (spur-like), broad, maximum width occupying about one-third of metatibial outer surface width (Fig. 20); second metasomal sternum with two narrow cowled slits.

## Discussion

As alluded to, while proposing the first comprehensive infrageneric classification for the genus *Euglossa*, Dressler (1978) created *Dasystilbe* as a monotypic subgenus to accommodate *Euglossa villosa*, which in his view shared features of bees included in two other subgenera. In this way, *Dasystilbe* represented an annectant taxon in Dressler’s system, bridging the divide between classificatory units as then proposed. In accordance with Dressler’s notion, it is true that the external morphology of *Euglossa villosa* exhibits features similar to *Glossura* and *Euglossella*. As already recognized by Dressler (1978), the facial and prothoracic features of *Euglossa villosa* resemble those of *Euglossella*, while the distinctive sternal cowled slits of the species are also a feature of several of the most common species of *Glossura*. The morphology of the male genitalia of *Euglossa villosa* similarly reveals a mosaic of characters, while features of the gonostylus clearly link it with *Glossura*, but most features of the hidden sterna are distinctly not as in *Glossura* (e.g., Hinojosa-Díaz 2008). As a monotypic entity in the classificatory scheme adopted for *Euglossa* by most researchers [i.e., the one established by Dressler (1978), and emended by the same author (Dressler 1982) and Moure (1989)], *Dasystilbe* is considered as having a rather stable position. However, these schemata lack phylogenetic validation, meaning that it is possible that *Dasystilbe* could be nested within another group. A molecular phylogeny for orchid bees and including a robust sampling of species from the genus *Euglossa* was recently completed (Ramírez et al. 2010). This study recovered *Euglossa villosa* as sister to *Euglossella* and together as sister to the remainder of the species in the genus. Morphological features that have historically been used to support such a placement include the strong clypeal ridges [which in recent analyses are highly homoplastic and of little phylogenetic value: Hinojosa-Díaz (2010, in prep.)], the pointed pronotal dorsolateral angle, the shape of the mesoscutellum (but, like the clypeal ridges, this same shape occurs in *Euglossella* and some species groups of *Glossura* and so its utility is unclear), the inflated and relatively smooth metatibia, and, most significantly, the morphology of the mesotibia, including the tufts. By contrast, a phylogenetic analysis conducted by Hinojosa-Díaz (2010, in prep.), based on morphology of the males and emphasizing numerous new genitalic characters, recovered *Euglossa villosa* as sister to a derived and redefined clade of *Glossura*. Curiously the results of both analyses reinforce the conflictive ideas on which Dressler based the erection of *Dasystilbe*, with each study supporting one of the two subgenera to which the species is purported to be allied. Regardless of the incongruent phylogenetic position of *Euglossa villosa* in both analyses, it is not nested within another subgenus as they are presently employed. Indeed, some additional external morphological attributes, not mentioned in the original description of *Euglossa villosa* by Moure (1968), are important as diagnostic features highlighting its distinctiveness from all other species of *Euglossa*, and provide additional insight into its phylogenetic placement. Aside from the gonostylar morphology, which is very close to that of some derived clades within the genus (i.e., *Euglossa* s.str. and *Glossura*) and totally dissimilar to that of *Euglossella*, the morphology of the second mesotarsomere is also shared with more derived clades. All other orchid bee genera have a generalized, unmodified second mesotarsomere, and this is certainly true for *Euglossella*, with the remainder of *Euglossa* s.l. having a basal emargination on the anterior margin of this tarsomere. Under the homology interpretation of Hinojosa-Díaz (2010, in prep.), *Euglossa villosa* has this emargination as well as a posterior basal emargination on the same podite unit (Figs 17–18). This feature is just another one of the many modifications in the legs of male orchid bees (particularly *Euglossa*) related to the handling of chemicals (e.g., Eltz et al. 2005) that seem to have had an impact on the diversification of the group.

## Supplementary Material

XML Treatment for Dasystilbe
